# Bibliography

**Published:** 1902-09-15

**Authors:** 


### BIBLIOGRAPHY.

CARE OF THE TEETH.—A little work of 150 pages by Dr. Samuel A. Hopkins, of Boston, on the "Care of the Teeth," has just come from the press of D. Appleton & Co. The object of the book is to indicate practicable methods by which diseases of the teeth may be prevented. The book is directed primarily to the application of sanitary measures in the early life of children. It calls attention first to the object of the teeth; then to their diseases and the causes. Proper food and exercise are very properly mentioned as the essential elements in dental prophylaxis. Then the mouth toilet is given its proper attention. The work is not a technical treatise, but is written for popular reading and is one of the best books of its kind we have ever examined. It can be most consistently recommended, especially to intelligent mothers, nurses and others having the care of children.

